# Editorial: New Strategies to Inhibit Cell Death in Myocardial Ischemia-Reperfusion Injury: How to Succeed?

**DOI:** 10.3389/fcvm.2022.918902

**Published:** 2022-07-04

**Authors:** Stéphanie Barrère-Lemaire, Christophe Piot, Sarawut Kumphune

**Affiliations:** ^1^IGF, Université de Montpellier, CNRS, INSERM, Montpellier, France; ^2^Département de Cardiologie Interventionnelle, Clinique du Millénaire, Montpellier, France; ^3^Biomedical Engineering Institute, Chiang Mai University, Chiang Mai, Thailand

**Keywords:** cell death, ischemia-reperfusion injury, myocardium, cardioprotection, diabetes, aging, therapeutics

## Introduction

Despite an active research activity since more than 30 years, there is no specific treatment nowadays against myocardial ischemia-reperfusion (IR) injury. Development of strategies based on inhibition of cell death appeared as a main issue for the reduction of injury to provide cardioprotection in the myocardial tissue. However, despite numerous putative drugs identified in animal models, no one of potential clinical utility has emerged. What would be the new ways to follow for discovering novel strategies of cardioprotection after a heart attack?

## I-Multiple Targets Therapies

Among cardioprotective strategies under investigation, cell therapy using various types of stem/stromal cells fully responds to the concept of a therapy based on a pleiotropic effect to fight multifaceted cardiac injury including cell death, inflammation and fibrosis. Mesenchymal Stromal/Stem Cell (MSC)-based therapy has been reported to improve the functional recovery of the ischemic myocardium by promoting endogenous cell survival, proliferation and angiogenesis. In particular, Shi et al. reports that neovascularization is the main mechanism of MSCs to improve the status of ischemic hearts. Blood supply being fundamental for the survival and the function of the myocardium, the formation of an efficient vascular network is a prerequisite for restoring durably the blood flow. MSCs activated by the hypoxic environment are able to differentiate into pericytes, endothelial, and smooth muscle cells. They regulate both angiogenesis and vasculogenesis through paracrine factors secreted throughout the neovascularization process (Shi et al.). Secretion of paracrine factors, rather than the differentiation process, is the main mechanism of action also evidenced in endothelial progenitor cells (EPCs) for their cardioprotective effects. Current knowledge on exosomes of EPC as putative therapeutic agents for treating cardiovascular disease as well as their mechanism of action is reviewed by Zeng et al..

For the treatment of multifaceted IR injury, MSC represent a lead candidate cell type during myocardial infarction. Nernpermpisooth et al. report that the cardioprotective effect of bone marrow-derived MSC depends on the presence of PPARβ/δ (*Peroxisome proliferator-activated* β*/*δ) receptors reported to play key roles in metabolism, angiogenesis and cell survival. In addition, they show using genetically modified MSC knockout for PPARβ/δ that the acute cardioprotective effect of MSC injected in the coronary network of *ex vivo* ischemic hearts is not related to their anti-inflammatory properties.

MSC therapeutic effects associated to their ephemeral presence in the IR myocardium suggests that cardioprotection is mediated through the release of paracrine factors such as extracellular vesicles (EV) that contain a variety of bioactive components able to rapidly educate immune cells and to protect cardiac cells. Zhang et al. report for the first time the expression profile of circular RNA involved in the cardioprotective effect mediated by EV derived from Human Umbilical Cord MSC (HuMSC) on cultured cardiac cells subjected to hypoxia-reoxygenation (HR). HuMSC-EVs treatment increases their survival rate due to the high level of expression of 10 circular RNA (circRNA) identified by High throughput RNA sequencing. GO (*Gene ontology*) and KEGG (*Kyoto Encyclopedia of Genes and Genomes*) pathway analyses allowed identifying that these circRNAs were related to important biological functions including cellular response to hypoxia and that the VEGF signaling pathway could be a mediator.

Although several small chemical compounds targeting cell death have been developed as potential therapeutic drugs, alternative medicine using natural extract from herbs, plant, or foods has also been explored and tested in preclinical models. One important issue is that such medicine is currently used in human. One of the major mechanisms of the natural phytochemical compounds is scavenging reactive oxygen radicals (ROS), which can effectively inhibit cellular damage at different levels. Chen et al. performed constructive review of several promising therapeutic candidate phytochemical compounds for myocardial IR injury. Several studies in different study models of myocardial IR injury were performed. Cardioprotection by phytochemical compounds is due to their anti-oxidant, anti-inflammatory, and anti-apoptotic effects. Several compounds were reported to downregulate cell signaling pathways involved in cell death such as ERK, p38, JNK, JAK/STAT, NF-κB, apoptotic regulatory p53, apoptotic regulatory Bcl-2/Bax proteins, and by contrast, to stimulate cell survival kinases including PI3K/Akt/GSK-3β. The original contribution from Givre et al. describes *in vitro* experiments on cardiac myocytes to evaluate the cardioprotective effects of hibernating bear serum against HR injury. The study shows that cell death inhibition was specific to the serum from brown bears, in contrast to horse or rabbit serum. The discovery of serum molecules coming from hibernating animals in non-hibernating animals opens a new therapeutic avenue for identifying cardioprotective molecules with future applications in humans (Givre et al.).

## II-Pharmacological Therapies

Since the discovery of small non-coding micro RNAs (miRNAs) in 1993, their roles and effect have been studied both in the regulation of normal physiological conditions and in pathogenesis. Wang and Zheng reviewed the protective role of miRNA against cellular apoptosis in myocardial IR injury as well as in post-ischaemic remodeling. Moreover, the article highlighted the relevance of targeting regulatory molecules of miRNA expression as alternative potential therapeutic strategies against myocardial IR injury (Wang and Zheng).

As another pharmacological approach, Parra-Flores et al. demonstrated that stimulation of Toll-Like Receptor (TLR) 4 could attenuate (simulated IR protocol) cardiac fibroblast cell death *in vitro via* activation of Akt and ERK survival kinases, suggesting that TLR4 could possibly be a novel therapeutic target to prevent cardiac cell death.

Proteins and peptides could also act as therapeutic agents to protect the myocardium. Pilose antler polypeptide (PAP-3.2KD) is one of the main active components from the traditional Chinese Medicine Pilose antler, known for its benefits in cardiomyopathy. Xu et al. demonstrated the protective effects of PAP against Adryomycin-induced myocardial injury mainly by inhibiting apoptosis. Recently, Li et al. demonstrated that the herbal extract Sweroside could protect cardiac cell death from IR-induced pyroptosis *via* a novel mechanism based on Kelch-like ECH-associated protein 1 (Keap1) inhibition and induced nuclear factor E2-associated factor 2 (Nrf2) nuclear translocation, which is a pathway involved in inflammation-related cell death.

A review article from Fernandez Rico et al. describes all the peptides that have been developed to treat myocardial IR injury. Therapeutic peptides could target apoptosis, necroptosis and inflammation activated during IR injury and some have been evaluated also in clinical trials. The authors present also their optimization in terms of targeting the ischemic area to limit off targets (Fernandez Rico et al.).

## III-Non-Pharmacological Therapies

Nowadays, non-pharmacological therapeutic strategies have also been evaluated and implemented to reduce the aggravation of myocardial IR injury. One of the well-known strategies to reduce cellular injury is based on lowering temperature or hypothermia. This technique has been proven to successfully reduce cardiac injury and infarct size, as well as improve cardiac function, not just only in experimental animal but also in clinical trials. Yamada et al. reviewed the implementation of therapeutic hypothermia in several study models. The gap of knowledge, which is related to the efficiency and safety of the technique, was also intensively identified. Another interesting article by Wang et al. introduces the physical cardioprotective strategy by electroacupuncture. An *in vivo* experiment is laboratory animals shows that electroacupuncture could precondition the heart by reducing cardiac cell injury and infarct size. The possible explanation of cardioprotection is based on apoptosis inhibition and survival kinases activation. However, several factors concerning safety need to be intensively investigated before proceeding to clinical applications.

## IV-Consideration of Comorbidities and Treatments

Until now, the translation of cardioprotection from animal experiments to randomized clinical trials has been rather disappointing. Unfortunately, there is a gap to be overcome and for that it is necessary to consider several elements that prevent from success.

First, most preclinical studies are performed in rodents animal models, as published in the collection “*New Strategies to Inhibit Cell Death in Myocardial Ischemia-Reperfusion Injury: How to Succeed*?.” A mandatory step toward clinical translation would be to repeat the experiments in a large species, most often the pig, as recommended by the working groups for the development of cardioprotective therapeutics, in order to confirm the results (1).

Second, laboratory animals are very different, in terms of pathophysiological conditions, from patients at risk of infarction. Rodents used in experimental studies are generally healthy young adults who differ from AMI patients with an average age of about 62 years as reported in most clinical studies. Aging is reported to impact on the increased susceptibility of cardiac cells to IR injury and could affect the effectiveness of cardioprotective strategies [see (2) for review].

Comorbidities such as diabetes and medications used in patients with acute myocardial infarction may also blind the beneficial effects of cardioprotective strategies studied in young and healthy animals. To overcome this critical issue, new experiments need to use more clinically relevant animal models.

Dia et al. compared the impact of type 2 diabetes on infarct size between STEMI patients and mice. They found that diabetic mice had larger infarcts than nondiabetic mice. However, they observed no difference between the two groups of patients, highlighting the fact that the diabetic patients were all treated with antidiabetic drugs, mainly Metformin. The authors also showed that Metformin was able to prevent an increase in the rate of cell death associated with the diabetes phenotype cultured cardiomyocytes following a hypoxia-reoxygenation protocol. treatment. Yu et al. also demonstrated that Dapagliflozin, a new type of antidiabetic medication that inhibit the sodium-glucose co-transporter-2, significantly improves ischemia-reperfusion induced cell death in non-diabetic mice by the selective autophagy degradation of the inflammasome component NLRP3.

The hallmarks of myocardial aging may also account for the discrepancy between animal and clinical studies. In a mini-review, Díaz-Vesga et al. provide un update concerning potential new cardioprotective strategies that could be used for the treatment of aging hearts.

## Conclusion

In order to ensure the successful development of new strategies to treat patients with AMI, it is important to consider drugs with pleiotropic effects acting (i) different pathways or (ii) different cell types, or by (iii) an improved tissue or subcellular targeting. Also, the therapeutical time window should be considered as well as aging, comorbidities and associated medicine treatments. The combination of these strategies should provide advantages for future clinical outcomes.

## Author Contributions

SB-L wrote the outline, introduction, conclusion, and the paragraph on cell therapy in Chapter I as well as Chapter II on pharmacological therapies concerning peptidic and protein tools. CP wrote Chapter IV on comorbidities and treatment options. SK wrote the paragraph on phytochemicals and traditional Chinese medicine in Chapter II and the paragraph on non-pharmacologic therapies in Chapter II. SB-L and SK reviewed, shortened, and edited the manuscript. All authors contributed to the article and approved the submitted version.

## Conflict of Interest

The authors declare that the research was conducted in the absence of any commercial or financial relationships that could be construed as a potential conflict of interest.

## Publisher's Note

All claims expressed in this article are solely those of the authors and do not necessarily represent those of their affiliated organizations, or those of the publisher, the editors and the reviewers. Any product that may be evaluated in this article, or claim that may be made by its manufacturer, is not guaranteed or endorsed by the publisher.

## References

[1] Jones SP Tang XL Guo Y Steenbergen C Lefer DJ Kukreja RC. The NHLBI-sponsored Consortium for preclinicAl assESsment of cARdioprotective therapies (CAESAR): a new paradigm for rigorous, accurate, and reproducible evaluation of putative infarct-sparing interventions in mice, rabbits, and pigs. Circ Res. (2015) 116:572–86. 10.1161/CIRCRESAHA.116.305462 25499773 PMC4329104

[2] Ruiz-Meana M Bou-Teen D Ferdinandy P Gyongyosi M Pesce M Perrino C. Cardiomyocyte ageing and cardioprotection: consensus document from the ESC working groups cell biology of the heart and myocardial function. Cardiovasc Res. (2020) 116:1835–49. 10.1093/cvr/cvaa132 32384145

